# Gamma irradiation mediated production improvement of some myco-fabricated nanoparticles and exploring their wound healing, anti-inflammatory and acetylcholinesterase inhibitory potentials

**DOI:** 10.1038/s41598-023-28670-5

**Published:** 2023-01-30

**Authors:** El-Sayed R. El-Sayed, Doaa S. Mansour, Reham M. Morsi, Hanan A. Abd Elmonem

**Affiliations:** 1 Plant Research Department, Nuclear Research Center, Egyptian Atomic Energy Authority, Cairo, Egypt; 2 Biological Applications Department, Nuclear Research Center, Egyptian Atomic Energy Authority, Cairo, Egypt

**Keywords:** Biochemistry, Biological techniques, Biotechnology, Microbiology, Nanoscience and technology

## Abstract

In the current scenario, scaling up the microbial production of nanoparticles with diverse biological applications is an emerging prospect for NPs’ sustainable industry. Thus, this paper was conducted to develop a suitable applicative process for the myco-fabrication of cobalt-ferrite (CoFeNPs), selenium (SeNPs), and zinc oxide (ZnONPs) nanoparticles. A strain improvement program using gamma irradiation mutagenesis was applied to improve the NPs-producing ability of the fungal strains. The achieved yields of CoFeNPs, SeNPs, and ZnONPs were intensified by a 14.47, 7.85, and 22.25-fold increase from the initial yield following gamma irradiation and isolation of stable mutant strains. The myco-fabricated CoFeNPs, SeNPs, and ZnONPs were then exploited to study their wound healing, and anti-inflammatory. In addition, the acetylcholinesterase inhibition activities of the myco-fabricated NPs were evaluated and analyzed by molecular docking. The obtained results confirmed the promising wound healing, anti-inflammatory, and acetylcholinesterase inhibition potentials of the three types of NPs. Additionally, data from analyzing the interaction of NPs with acetylcholinesterase enzyme by molecular docking were in conformation with the experimental data.

## Introduction

Biogenic nanoparticles (NPs) are gaining great importance for their applications in several medical and industrial sectors^1^. Among the emerging biogenic NPs, Cobalt-ferrite (CoFeNPs), Selenium (SeNPs), and Zinc oxide (ZnONPs) possess several unique properties including their biocompatibility that make them emerging candidates for many biological applications^2–4^. Recent progress of these NPs in several sciences arose from their unique shape, size, composition, and crystallinity-dependent chemical, physical, and biological characteristics^5–7^. In recent years, the production of NPs using microorganisms provided several advantages over other techniques and is increasingly being explored^8^. In this regard, the microbial biosynthesis of CoFeNPs, SeNPs, (by *Monascus purpureus*), ZnONPs (by *Alternaria tenuissima*) and their physical characterization as well as their biological activities have been previously demonstrated^2–4^. As a part of the continuing research work in this concern, we aim in this paper to further intensify the biosynthetic potential of these fungal cultures using gamma-irradiation. Gamma irradiation mutagenesis of the fungal strains involved in the preparation of the three types of NPs aim to enhance the productivity process and develop stable mutant strains for the first time for more effective hyperproduction of CoFeNPs, SeNPs, and ZnONPs.

Previous reports concerning the myco-fabricated CoFeNPs^2^, SeNPs^3^, and ZnONPs^4^ revealed excellent biological activities including antibacterial, anticancer, antifungal, and antioxidant. Taking into consideration the emerging developments in the use of these NPs, we aim in this paper to further explore their wound healing, anti-inflammatory, and acetylcholinesterase inhibitory potentials. Wound healing is the process of restoring damaged tissues^9^. It contains four sequential steps including hemostasis^10^, inflammation^11^, proliferation, and remodeling^12^. Due to the emergence of multidrug-resistant microbes that delay the healing process^9^, there is a pressing need to develop novel non-toxic materials to improve the efficacy of the wound-healing process^12^. Inflammation is an essential defense system in the human body to fight infections^13^. Nevertheless, uncontrolled inflammation could cause several diseases including arthritis^14^, cardiovascular, cancer^15^, genetic^16^, and neurodegenerative diseases^17^.

Alzheimer's is a progressive neurodegenerative disease that presents the most common form of dementia; affects thinking, memory, and behavior^18^. Till now, there is no effective therapeutic procedure for the disease, so it is a priority area for research to find effective therapeutics. Recently, cholinesterase inhibitors present a popular target^19^ where the levels of acetylcholine and butyrylcholine are maintained by inhibition of the enzyme. Cholinesterase enzyme can hydrolyze the neurotransmitter acetylcholine and is one of the most crucial enzymes for nerve function and response^20^. Little work has been done to investigate the interactions between the acetylcholinesterase enzyme and the three types of NPs^18^. Thus, the presented research was extended to evaluate the in vitro wound healing, anti-inflammatory, and acetylcholinesterase inhibitory potentials of CoFeNPs, SeNPs, and ZnONPs.

## Materials and methods

### Synthesis, purification, and characterization of CoFeNPs, SeNPs, and ZnONPs

The three types of NPs viz., CoFeNPs, SeNPs, and ZnONPs were prepared by the extracellular mycosynthesis technique. The fungus *Monascus purpureus* ATCC16436 was used to synthesize CoFeNPs and SeNPs according to the methods described in detail in previous studies^2,3^. Meanwhile, ZnONPs were synthesized according to a previous study^4^ of the fungus *Alternaria tenuissima* AUMC10624. In brief, the two fungal strains were separately cultured in potato-dextrose broth and the obtained cell-free culture filtrates were mixed with an aqueous solution of the corresponding salt (cobalt nitrate and ferric nitrate in a 1:2 M for CoFeNPs, 1 mM sodium selenite for SeNPs, and 2 mM zinc sulfate for ZnONPs). Then, the mixtures from each salt were vigorously stirred for 20 min kept at room temperature and the precipitate from each mixture was separated by ultracentrifugation. Each precipitate was washed in deionized water followed by ethanol and finally dried at 50 °C. The obtained fine powder from every precipitate was dissolved in HPLC ethanol and treated ultrasonically for the dispersion of NPs. Characterization of the obtained NPs was accomplished by X-ray diffraction, Zeta potential and Dynamic light scattering (DLS) analyses, and Transmission Electron Microscope, according to previous reports^2–4^.

### Gamma irradiation mutagenesis

To induce mutants with improved productivities of CoFeNPs, SeNPs, and ZnONPs, spore suspensions of *Monascus purpureus* ATCC16436 and *Alternaria tenuissima* AUMC10624 were separately prepared. According to previous studies^2,3,21^, 1000 Gy (for CoFeNPs and SeNPs) and 500 Gy (ZnONPs) of gamma rays were the most proper doses for maximum production of these NPs, so we used the same irradiation doses in this paper to isolate mutant strains with improved productivities. The irradiation process was performed using a ^60^Co Gamma chamber (MC20, Russia) at the Nuclear Research Center, Egyptian Atomic Energy Authority. After irradiation, all the exposed suspensions were stored at 4 °C in darkness overnight. After which, the irradiated suspensions were diluted, and 50 μL was spread on potato-dextrose agar plates and then incubated at 25 °C for 7 days. The appeared colonies were collected and tested for their enhanced production of the three NPs. Finally, the highest NPs-producing mutant strains (*M. purpureus* Mutant-2022 and *A. tenuissima* Mutant-1985) were followed up for their stability across six successive generations, under the conditions described earlier. The yield of NPs was estimated by weighing the obtained powders from every irradiation dose and expressed as mg NPs mL^−1^ ^2,3,21^.

### In vitro wound healing potential

The wound healing potential of CoFeNPs, SeNPs, and ZnONPs was studied against human dermal fibroblast (HDF) cell lines by the wound scratch assay test according to the previously reported and standardized protocols^22,23^. HDF cell lines were maintained in Dulbecco’s Modified Eagle’s Medium (Corning, USA) supplemented with 100 mg mL^-1^ of streptomycin, 100 units/mL penicillin, 10% heat-inactivated fetal bovine serum in humidified and incubated at 37 °C in a 5% (v/v) CO_2_ atmosphere. Cells were plated at a density of 3 × 10^5^/well onto a coated 6-well plate and cultured overnight. On the next day, horizontal scratches were introduced into the confluent monolayer with a sterile pipette tip and the plate was washed thoroughly with PBS. Control wells were replenished with fresh medium only while treated wells were replenished with fresh media containing either CoFeNPs, SeNPs, or ZnONPs at a concentration of 100 μg mL^−1^. The plate was incubated at 37 °C and 5% CO_2_. The distance between two layers of the scratched cells was then inspected microscopically at 0, 24, 48, 72, and 96 h. As the HDF cells migrate to fill the scratched area, images were captured by a digital camera attached to a microscope and computer system. The acquired images were analyzed by MII ImageView software version 3.7.

### In vitro anti-inflammatory potential

Murine macrophages RAW264.7 cells (ATCC) were maintained in Dulbecco’s Modified Eagle’s Medium (Corning, USA) supplemented with fetal bovine serum (10%), streptomycin sulfate (100 µg mL^−1^), penicillin (100 U mL^−1^), and 2 mM L-glutamine in a humidified 5% CO_2_ incubator. For passaging and treatment, cells were washed with PBS and scrapped off the flasks using sterile scrappers (SPL, Spain). RAW 264.7 cell stock (0.5 × 10^6^ cells/mL) was seeded into 96-well microwell plates and incubated overnight. On the next day, non-induced triplicate wells received medium only. The inflammation group of triplicate wells received the inducer of inflammation (lipopolysaccharide (LPS) as 100 ng/mL in complete culture media). Sample groups of triplicate wells received final concentrations (0.1–1000 µg/mL) of either CoFeNPs, SeNPs, or ZnONPs using PBS as a vehicle and diluted into culture media containing LPS. Indomethacin (Indo, 0.25 mM) was used as an anti-inflammatory positive control. After 24 h of incubation, the Griess assay was used to determine nitrite (NO) production in all wells^24^. Culture supernatants and Griess reagent were mixed (equal volume basis) and incubated at room temperature for 10 min to form the colored diazonium salt and read at an absorbance of 540 nm on a Tecan Sunrise™ microplate reader (Austria). NO inhibition (%) was calculated relative to the LPS-induced inflammation group, normalized to cell viability determined with Alamar Blue™ reduction assay^25^.

### In vitro acetylcholinesterase inhibition

The acetylcholinesterase inhibitory potential of CoFeNPs, SeNPs, and ZnONPs was determined by Ellman’s assay^26^ modified by Gorun and co-authors^27^ in a crude preparation. NPs were separately dissolved in phosphate buffer at a concentration range of 0.1–1000 µg mL^−1^. The acetylcholinesterase enzyme source was a 5% w/v fresh rat brain homogenate in a phosphate buffer. The substrate was aqueous 1.0 mM acetylcholine iodide and the co-substrate was DTNB–phosphate–ethanol reagent. Galantamine hydrobromide was applied as a positive control. 100 μL of the NPs solution was transferred to the test tubes, adding 100 μL of the crude enzyme, and 150 μL of phosphate buffer and incubated at 37 °C for 15 min. Thereafter, 50 μL acetylcholine iodide was added and incubated again. Finally, 1.8 mL of the DTNB–phosphate–ethanol reagent was added to stop the reaction. The resultant color was immediately monitored by a UV spectrophotometer (Jenway 6305, UK) at 412 nm. The blank was a sample without an enzyme. The percent of inhibition was estimated by the following equation:

$$\% \;{\text{ of}}\; {\text{inhibition}}\, = \,\left( {{\text{C}}\, - \,{\text{E}}} \right)/{\text{C}}\, \times \,{1}00$$

where C is the control absorbance unit and E is the experimental absorbance unit.

### Molecular docking studies

A molecular docking tool was used to investigate the interactions between the acetylcholinesterase enzyme and the three types of NPs^28^. The acetylcholinesterase crystal (PDB codes: 1OCE) structure was obtained from the protein database bank (https://www.rcsb.org). Water molecules were removed at first from the complex and the crystallographic disorders and unfilled valence atoms were corrected using protein report and utility and clean protein options. Protein-energy was minimized by applying MMFF94 force fields. The rigid binding site was the structure of the protein obtained by applying a fixed atom constraint. The protein essential amino acids are defined and prepared for the docking process. 2D structures of CoFeNPs, SeNPs, and ZnONPs were drawn using Chem-Bio Draw Ultra17.0. Using AUTODOCK VINA Software, 3D structures were protonated, and energy was minimized by applying 0.05 RMSD kcal mol^-1^ MMFF94 force field. Then, the minimized structures were prepared for docking using the prepared ligand protocol. The molecular docking process was carried out using CDOCKER protocol. The receptor was held rigid while the ligands were allowed to be flexible during the refinement each molecule was allowed to produce ten different interaction poses with the protein. Then docking scores (-CDOCKER interaction energy) of the best-fitted poses with the active site at the acetylcholinesterase enzyme were recorded and a 3D view was generated by Discovery Studio 2019 Client software. All of these processes were used to predict the proposed binding mode, affinity, preferred orientation of each docking pose, and binding free energy (∆G) of CoFeNPs, SeNPs, and ZnONPs with the acetylcholinesterase enzyme.

### Statistics

Results were expressed as the mean (calculated from triplicate measurements from two independent experiments) ± standard deviation. Statistical significance between means was determined by the ONE-WAY ANOVA test followed by the Least Significant Difference test (at 0.05 level) using SPSS software (v 22, IBM Corp, NY).

## Results and discussion

### Synthesis and characterization of CoFeNPs, SeNPs, and ZnONPs

**Figure 1:**
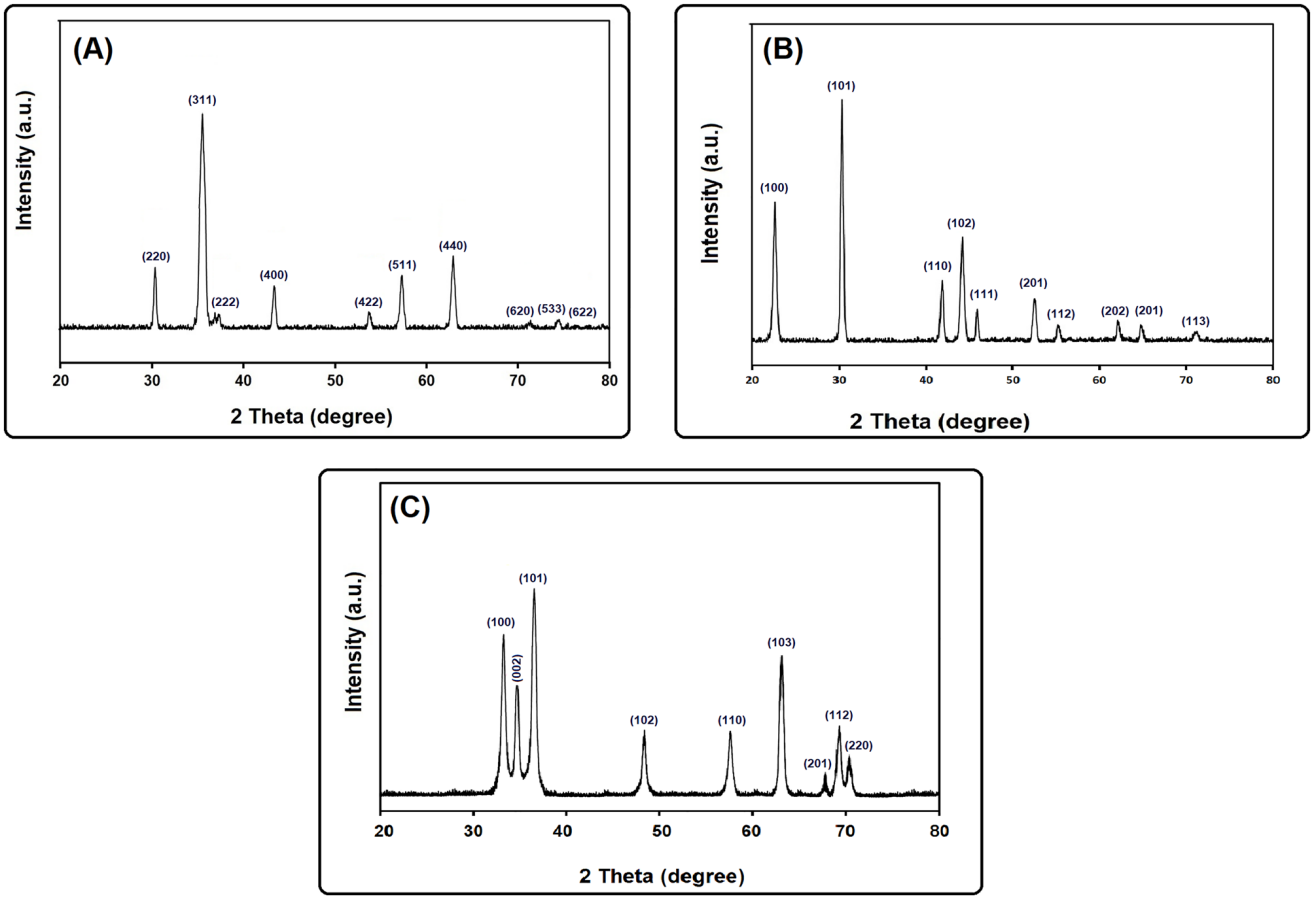
X-ray diffraction pattern (Cu Kα-radiation) at room temperature of the myco-fabricated CoFeNPs (**A**), SeNPs (**b**), and ZnONPs (**C**).

**Figure 2:**
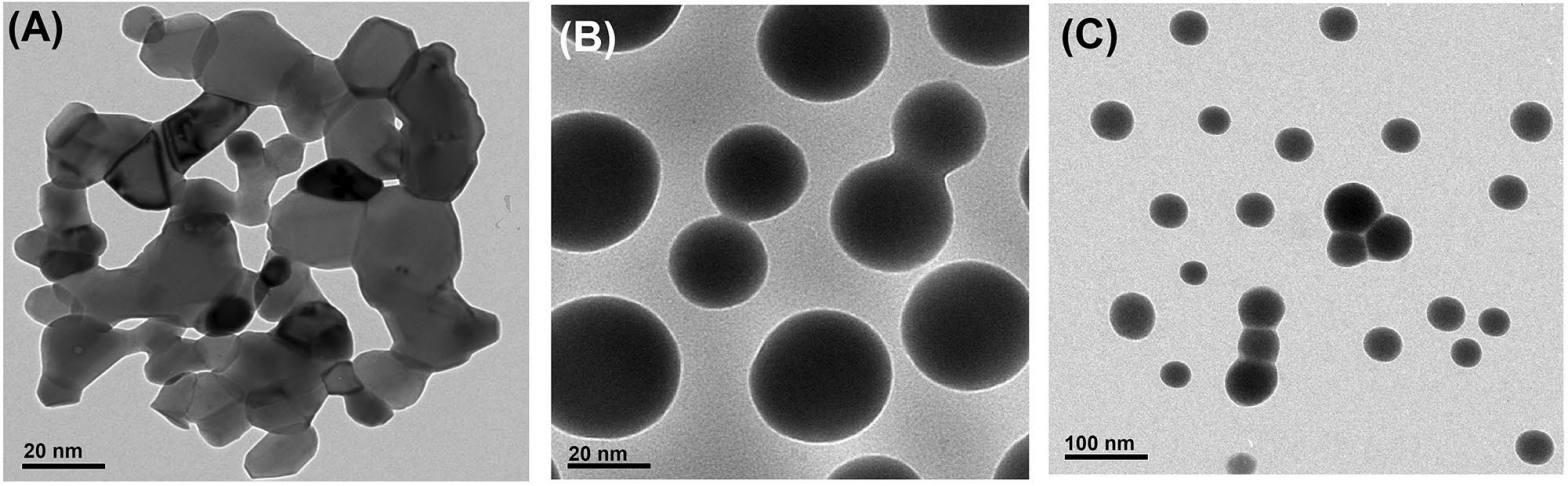
TEM images of the myco-fabricated CoFeNPs (**A**), SeNPs (**B**), and ZnONPs (**C**).

**Figure 3:**
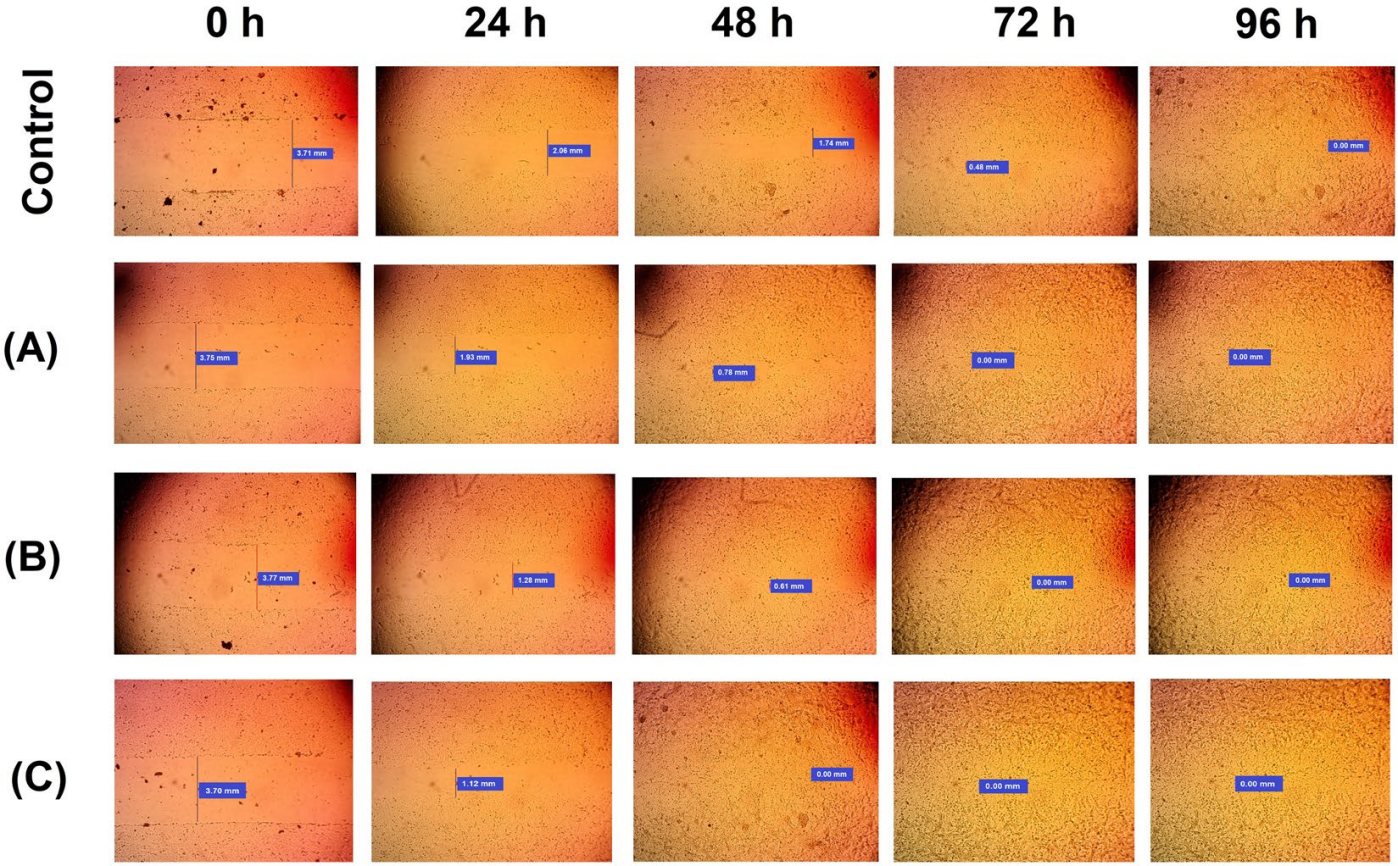
Wound healing potential of CoFeNPs (**A**), SeNPs (**B**), and ZnONPs (**C**).

Figure 1 and Table 1 show XRD patterns and crystallographic data of the myco-fabricated CoFeNPs, SeNPs, and ZnONPs. The obtained data for CoFeNPs (Fig. 1A) showed that the structure of the synthesized NPs was inverse cubic with a lattice parameter of 0.838140 nm which is in agreement with the standard card JCPDS No. 22–1086^2,29^. SeNPs (Fig. 1B) showed a hexagonal crystal structure with a lattice parameter of 0.445070 nm which is in agreement with the standard card JCPDS No. 06-0326^3,8^. XRD data of ZnONPs (Fig. 1C) showed that the crystal structure was hexagonal with a lattice parameter of 0.323110 nm which is in agreement with JCPDS card No. 361451^4,21,30^. It is noteworthy that, all the myco-fabricated NPs are pure and of a single phase (Fig. 1A, B, and C) where no peaks corresponding to impurities were detected. Table 1 also presents the crystallite sizes (calculated from the Scherrer equation from the FWHM of the most intense peak) of CoFeNPs (8.97 nm), SeNPs (48.76 nm), and ZnONPs (18.65 nm). DLS analysis (Table 1) showed that the recorded average sizes of the myco-fabricated CoFeNPs (7.65 nm), SeNPs (48.11 nm), and ZnONPs (16.72 nm), which are in agreement with those of the XRD analysis. The recorded PDI values were 0.243 for CoFeNPs, 0.255 for SeNPs, and 0.291 for ZnONPs. Their zeta potential values were –24.55 mV, –23.85 mV, and –21.63 mV, respectively. Interestingly, all the aqueous solutions of the myco-fabricated NPs showed excellent stability for 1 year. Figure 2 presents the morphology of the myco-fabricated NPs. TEM micrographs of CoFeNPs (Fig. 2A), SeNPs (Fig. 2B), and ZnONPs (Fig. 3C) confirmed their spherical shape. All of the observations regarding the crystallographic data, DLS data, and morphology of the three were in good agreement with previous studies concerning CoFeNPs^2^, SeNPs^3^, and ZnONPs^4,21^.

**Table 1:** Crystallographic data and DLS analysis of the myco-fabricated NPs.

Parameter	Myco-fabricated NPs		
	CoFeNPs	SeNPs	ZnONPs
Formula	CoFe_2_O_4_	Se	ZnO
Crystal system	cubic	hexagonal	hexagonal
Lattice parameter (nm)	0.838140	0.445070	0.323110
Mean crystallite size (nm)	8.97	48.76	18.65
Size distribution (nm)	5−15	15−65	15−60
Average size (nm)	7.65	48.11	16.72
Zeta potential (mV)	−24.55	−23.85	−21.63
Polydispersity index	0.243	0.255	0.291

The mean crystallite size was calculated from the Scherrer equation. Average size, size distribution, zeta potential, and Polydispersity index were obtained from the DLS analysis as described in Materials and Methods.

### Improvement of NPs’ yield by Co^60^ gamma irradiation mutagenesis

Here, we designed a stain improvement program to intensify the synthetic ability of* M. purpureus* and *A. tenuissima* for CoFeNPs, SeNPs, and ZnONPs. Taken the advantage of the previous reports concerning the two strains^2,3,21^. After irradiation, 65 mutants were isolated and separately screened for their enhanced productivities of the three types of NPs. Of these mutants, *M. purpureus* Mutant-2022 and *A. tenuissima* Mutant-1985 showed higher productivity than other mutants. So, we followed the production stability of the three types of NPs across six successive generations. Table 2 confirmed that the two mutants were stable mutants where no statistically significant differences (*P* ≤ *0.05*) were observed in the recorded yield of either CoFeNPs, SeNPs, or ZnONPs. The achieved yield of the respective NPs in this study attained 359.81, 476.62, and 1780.12 mg mL^−1^ which represent a 14.47, 7.85, and 22.25-fold increase from the initial yields of CoFeNPs (24.87 mg mL^−1^)^2^, (60.69 mg mL^−1^) SeNPs^3^, and (80.01 mg mL^−1^) ZnONPs^21^, respectively. In good agreement with our results, exposure of *A. terreus* spores to gamma radiation at the same dose resulted in improved production and high yield of five types of NPs^31^. In literature, several reports recommend gamma rays as a physical mutagen for the improvement of microbial cultures to intensify their production abilities^32–34^. It is noteworthy that, this improvement of microbial cultures using gamma irradiation mutagenesis could lead to the isolation of hyper-producers thereby lowering the overall cost of the process^35–38^.

**Table 2:** NPs yield (mg L^-1^ culture filtrate) of CoFeNPs and SeNPs synthesized by *M. purpureus* Mutant-2022 and ZnONPs synthesized by *A. tenuissima* Mutant-1985 mutant strains grown for six successive generations.

Generation	NPs yield (mg mL^-1^)		
	CoFeNPs	SeNPs	ZnONPs
First	356.87 ± 56.87^a^	458.98 ± 121.67^a^	1786.88 ± 108.56^a^
Second	362.98 ± 49.21^a^	492.66 ± 98.67^a^	1704.56 ± 137.72^a^
Third	358.88 ± 63.91^a^	448.67 ± 106.56^a^	1799.56 ± 121.67^a^
Fourth	376.87 ± 43.78^a^	467.89 ± 112.76^a^	1748.59 ± 100.65^a^
Fifth	359.67 ± 22.98^a^	482.78 ± 106.56^a^	1739.56 ± 128.67^a^
Sixth	359.81 ± 45.32^a^	476.62 ± 110.56^a^	1780.12 ± 135.87^a^

The calculated mean is for triplicate measurements from two independent experiments ± SD.

^a^means with different superscripts in the same column are considered statistically different (LSD test, *P* ≤ *0.05*).

### Wound healing potential of CoFeNPs, SeNPs, and ZnONPs

Table 3 presents the recorded data on wound healing potentials of the three NPs involved in measuring the initial wound width (0 h) along with healing towards wound closure (96 h). Generally, the wound was closed after 96 h for the control. The recorded wound width for control was 3.72 mm (0 h), 2.06 mm (24 h), 1.77 mm (48 h), and 0.47 mm (72 h). Wounds treated with CoFeNPs showed 3.75 mm (0 h), 1.96 mm (24 h), and 0.85 mm (48 h). Wounds treated with SeNPs displayed 3.77 mm (0 h), 1.29 mm (24 h), and 0.68 mm (48 h). Interestingly, wounds treated with ZnONPs displayed 3.70 mm (0 h), and 1.01 mm (24 h) (Table 3). The results further showcased a complete wound closure after treatment with CoFeNPs and SeNPs after 72 h (Fig. 3). Meanwhile, ZnONPs treated wounds had significantly the fastest healing effect, where complete wound closure was observed after 48 h. In the literature, several reports studied the wound-healing potential of metal NPs mainly silver, gold, zinc, titanium oxide, iron oxide, and copper NPs^39^. However, few reports focused on SeNPs^40,41^. It is noteworthy that the present study is the first report on using CoFeNPs for wound healing. Generally, metal NPs are more favorable than the traditional wound healing agents because they possess better intrinsic qualities, such as optical, catalytic, and melting properties^42^. The nano size and shape, surface properties, porosity, and the ability of metals to resist decomposition in aqueous solutions, contribute to their efficacy in biological applications^39–41^. Furthermore, metal NPs are growingly being used in dermatology, owing to their beneficial effects including wound healing acceleration, antimicrobial potential, less frequent dressing changes, and ease of use [42, and references therein].

**Table 3:** Wound healing potential of the myco-fabricated CoFeNPs, SeNPs, and ZnONPs.

NPs	Wound width (mm)				
	0 h	24 h	48 h	72 h	96 h
Control	3.72 ± 0.18^a^	2.06 ± 0.14^b^	1.77 ± 0.22^c^	0.47 ± 0.07^d^	00.00 ± 0.00^e^
CoFeNPs	3.75 ± 0.31^a^	1.96 ± 0.51^b^	0.85 ± 0.04^c^	00.00 ± 0.00^d^	00.00 ± 0.00^d^
SeNPs	3.77 ± 0.43^a^	1.29 ± 0.11^b^	0.68 ± 0.03^c^	00.00 ± 0.00^d^	00.00 ± 0.00^d^
ZnONPs	3.70 ± 0.21^a^	1.01 ± 0.25^b^	00.00 ± 0.05^c^	00.00 ± 0.00^c^	00.00 ± 0.00^c^

The calculated mean is for triplicate measurements from two independent experiments ± SD.

^a–e^means with different superscripts in the same row are considered statistically different (LSD test, *P* ≤ 0.05).

### Anti-inflammatory activity of CoFeNPs, SeNPs, and ZnONPs

During the progress of inflammation, several growth factors and inflammatory mediators are produced. Among these mediators, nitrite (NO) is evidenced to be a basic inflammatory component in the development of inflammation^43–45^. Previous reports have suggested that the majority of inflammatory diseases are caused by the up-regulation of NO production [^46^, and references therein]. Besides, the down-regulation of NOS is an important therapy for the proper treatment and prevention of inflammatory diseases. Accordingly, we evaluated the inflammatory inhibition ability of CoFeNPs, SeNPs, and ZnONPs by nitrite oxide assay as a result of NO production. Our results (Table 4) confirmed that CoFeNPs, SeNPs, and ZnONPs inhibited the LPS-induced production of NO in a dose-dependent manner. Furthermore, our results indicated the superiority of SeNPs over CoFeNPs and ZnONPs. The recorded minimum inhibitory concentration for SeNPs was at 0.1 µg mL^−1^, while it was at 10 µg mL^−1^ for both CoFeNPs and ZnONPs. Thus, these findings suggest that the three types of NPs have anti-inflammatory effects by inhibiting the secretion of the pro-inflammatory mediator NO in cells stimulated by LPS. In accordance with our results, El-Ghazaly and co-workers studied the anti-inflammatory potential of SeNPs on inflammation induced in rats exposed to radiation^47^. The authors concluded that SeNPs were effective in reducing the paw volume in non-irradiated and irradiated rats but it did not alter the nociceptive threshold. Moreover, SeNPs decreased the elevation of all the estimated parameters caused by the induced inflammation in non-irradiated and irradiated rats^47^. Our study is the first report on the anti-inflammatory potential of CoFeNPs, despite their importance in cancer treatment due to their magnetic properties. Meanwhile, the reduction of protein expression levels of LPS-induced NOS by ZnONPs has been recorded in a dose-dependent manner^48^. In another study, ZnONPs at a concentration of 10 μg mL^−1^ inhibited the production of NO induced by LPS in RAW 264.7 macrophages^49^. Generally, NO is a cell-signaling free radical that can freely diffuse across cell membranes and significantly affect the pathogenesis of many inflammatory disorders^50^. It also showed a significant role in regulating different inflammatory events including pro-inflammatory gene transcription^46^, leukocyte rolling and transmigration^50^, and regulation of vascular responses^51^.

**Table 4:** Effects of CoFeNPs, SeNPs, and ZnONPs on NO inhibition (%) in RAW264.7 murine macrophages.

NPs concentration (µg mL^-1^)	NO Inhibition (%)			
	Control	CoFeNPs	SeNPs	ZnONPs
0.00 (C)	0.00^e^	0.00^d^	0.00^e^	0.00^d^
0.1	19.89 ± 1.21^d^	0.00^d^	8.59 ± 2.39^d^	0.00^d^
10	39.41 ± 2.17^c^	48.21 ± 2.22^c^	59.81 ± 1.51^c^	36.43 ± 1.44^c^
100	75.77 ± 3.54^b^	59.89 ± 0.94^b^	86.41 ± 2.38^b^	69.41 ± 4.21^b^
1000	98.69 ± 1.82^a^	70.89 ± 2.78^a^	91.43 ± 1.07^a^	79.51 ± 2.76^a^

Indomethacin was used as the positive control. Macrophages RAW264.7 cells were pre-treated and then stimulated with LPS. The concentrations of Nitrite were determined as described in the materials and methods. The calculated mean is for triplicate measurements from two independent experiments ± SD.

^a–e^means with different superscripts in the same column are considered statistically different (LSD test, *P* ≤ 0.05).

### Acetylcholinesterase inhibitory potential of CoFeNPs, SeNPs, and ZnONPs

Table 5 presents the acetylcholinesterase inhibitory activities of CoFeNPs, SeNPs, and ZnONPs. Interestingly, the three types of NPs inhibited the acetylcholinesterase enzyme in a dose-dependent manner. The obtained data (Table 5) further indicated that ZnONPs had a stronger inhibitory effect on acetylcholinesterase than CoFeNPs and SeNPs. At a concentration of 1000 µg mL^−1^, ZnONPs showed 99.21% of inhibition, while at the same concentration it was 95.78 for CoFeNPs and 90.91% for SeNPs. These findings are noteworthy because Alzheimer's disease is associated with acetylcholinesterase enzyme deficiency and the three types of the myco-fabricated NPs could be potential new acetylcholinesterase inhibitors. In agreement with our results, several concentrations of zinc oxide, nickel oxide, iron oxide, lead oxide, and cobalt oxide were evaluated as acetylcholinesterase inhibitors^52^. The authors reported the same observation of the dose-dependent response of the inhibition of the enzyme by the different types of NPs. Moreover, the highest inhibition was shown by PbONPs^52^. In the same connection, Wang and co-workers concluded that increasing concentrations of NPs showed a gradual increase in the recorded activity inhibition^53^. In addition, the excellent inhibitory efficacy of AuNPs^54^ and AgNPs^55^ against acetylcholinesterase was reported. In the literature, there is no clear explanation of how the NPs interact with the enzyme. However, a previous report proposed that inhibition by NPs is primarily caused by interaction or adsorption with acetylcholinesterase protein^53^. In this regard, we used molecular docking to explore the mechanistic interaction between the three myco-fabricated NPs and acetylcholinesterase proteins.

**Table 5:** Acetylcholinesterase inhibitiory potential of CoFeNPs, SeNPs, and ZnONPs.

Concentration (µg mL^-1^)	Inhibition (%)			
	Control	CoFeNPs	SeNPs	ZnONPs
0.00 (C)	0.00 ± 0.00^e^	0.00 ± 0.00^e^	0.00 ± 0.00^e^	0.00 ± 0.00^e^
0.1	22.56 ± 0.45^d^	63.51 ± 0.51^d^	35.41 ± 0.47^d^	75.37 ± 0.56^d^
10	41.65 ± 0.21^c^	85.76 ± 0.77^c^	67.93 ± 0.56^c^	84.98 ± 0.82^c^
100	76.43 ± 0.33^b^	90.21 ± 0.59^b^	82.45 ± 0.92^b^	93.78 ± 0.57^b^
1000	90.47 ± 0.52^a^	95.78 ± 0.48^a^	90.91 ± 0.48^a^	99.21 ± 0.77^a^

Galanthamine hydrobromide was used as the positive control. The calculated mean is for triplicate measurements from two independent experiments ± SD.

^a–e^means with different superscripts in the same column are considered statistically different (LSD test, *P* ≤ 0.05).

### Molecular docking of NPs and acetylcholinesterase enzyme

**Figure 4:**
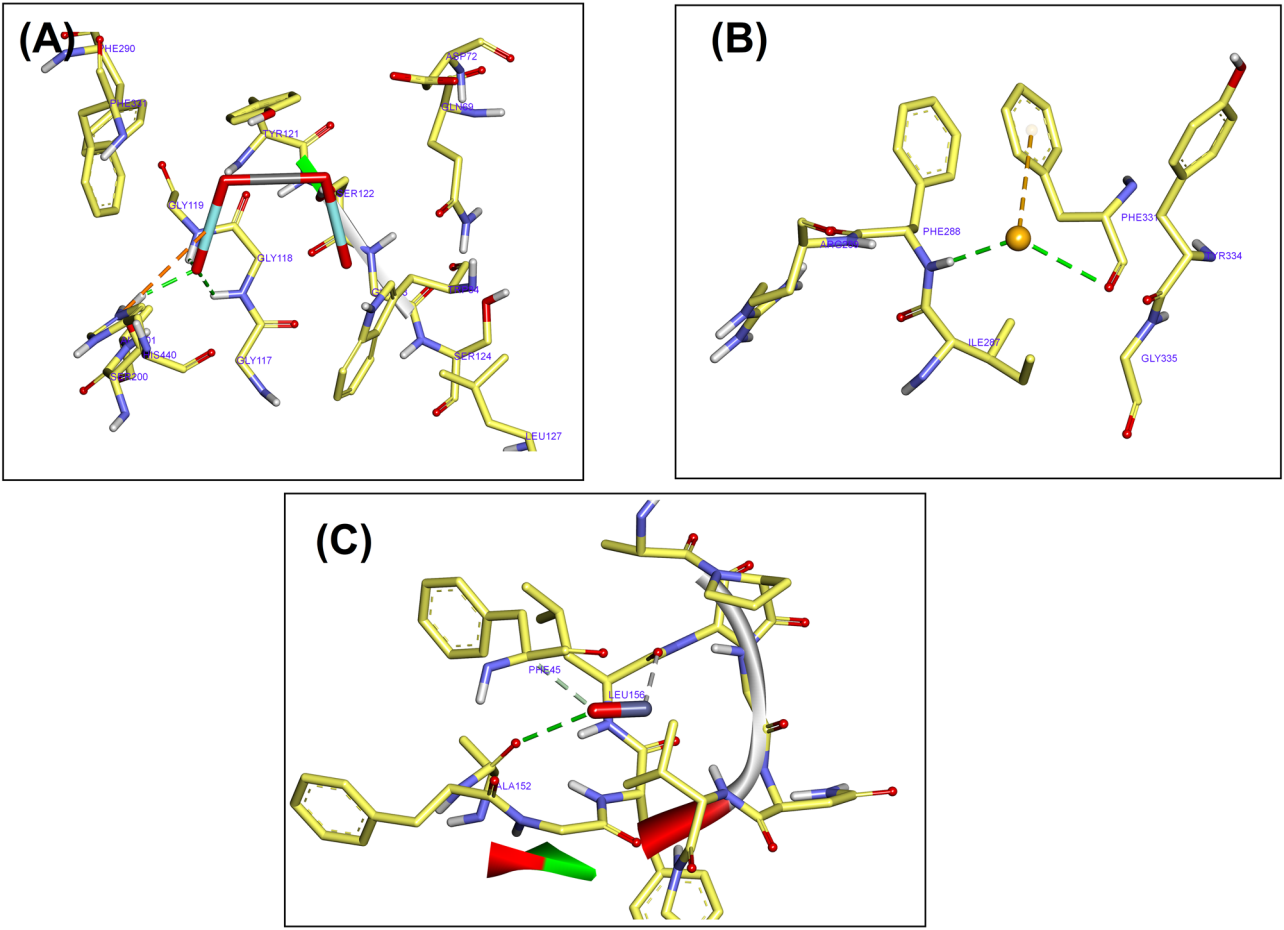
Docking conformation of CoFeNPs (**A**), SeNPs (**B**), and ZnONPs (**C**) in the active site of acetylcholinesterase enzyme.

**Figure 5:**
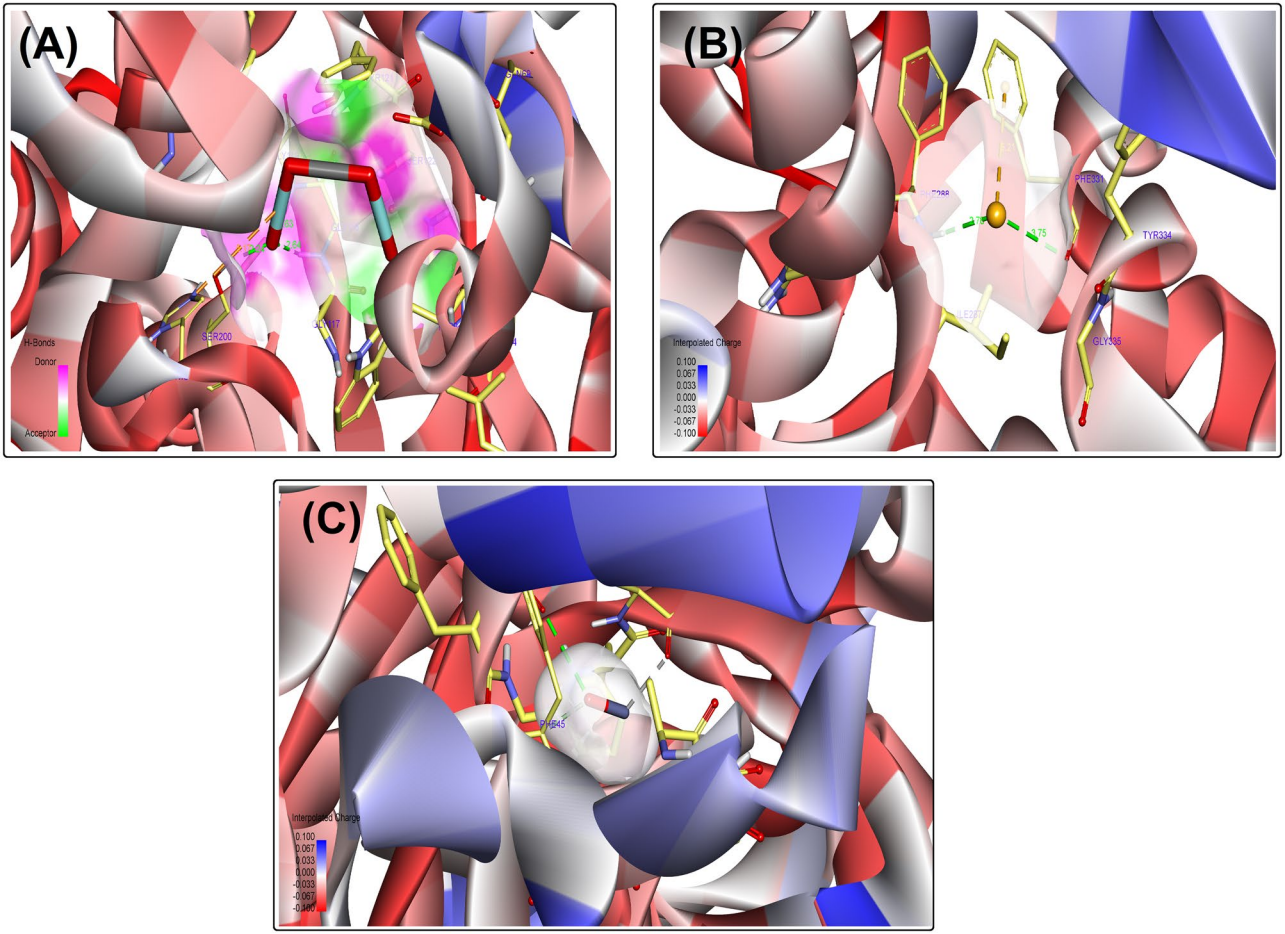
3D interaction of CoFeNPs (**A**), SeNPs (**B**), and ZnONPs (**C**) in the active site of acetylcholinesterase enzyme.

Figures 4 and 5 present the docking conformation of CoFeNPs, SeNPs, and ZnONPs in the active site of the acetylcholinesterase enzyme. The binding mode of CoFeNPs exhibited an energy binding of –4.42 kcal mol^−1^ against the acetylcholinesterase enzyme. CoFeNPs interacted with Gly118, Gly119, and Ser200 by three hydrogen bonds with a distance of 2.36, 2.64, and 2.24ºA. Additionally, it interacted by attractive charged interaction with His440 (Figs. 4A and 5A). The binding mode of SeNPs exhibited an energy binding of −2.36 kcal mol^−1^, which bind with Phe331 and Phe288 by hydrogen bonds with a distance of 2.46 and 1.64ºA. Besides, binding interaction with Phe331 (Figs. 4B and 5B). The binding mode of ZnONPs exhibited an energy binding of −3.56 kcal mol^−1^ against the acetylcholinesterase enzyme. ZnONPs are attached to Ala152 by a hydrogen bond with a distance of 2.85ºA. (Figs. 4C and 5C). In the literature, data regarding the molecular docking of NPs and acetylcholinesterase enzyme are rare. There is only one report on the molecular docking of some metal NPs against the enzyme^52^. The authors reported similar observations to our findings; NPs interacted with the residue Gly119, Tyr121, and Val293.

## Conclusion

We conclude that CoFeNPs, SeNPs, and ZnONPs could be efficiently produced by fungi at high yields. The achieved yield of the respective NPs in this study attained 359.81, 476.62, and 1780.12 mg mL^−1^ which represent a 14.47, 7.85, and 22.25-fold increase from the initial yields. The developed process is an applicative process for the myco-fabrication of the three NPs which will open the way towards their production on an industrial scale. Further studies on using bioreactors for the production process are needed. Moreover, the present study suggests the wound healing potential CoFeNPs for the first time. Besides, the promising anti-inflammatory and acetylcholinesterase inhibitory potentials of the three types of NPs. Our results indicated that ZnONPs had a stronger inhibitory effect on acetylcholinesterase and wound healing potential than CoFeNPs and SeNPs. ZnONPs treated wounds had significantly the fastest healing effect, where complete wound closure was observed after 48 h. Furthermore, our results indicated the superiority of SeNPs as an anti-inflammatory agent over CoFeNPs and ZnONPs in inhibiting the LPS-induced production of NO in a dose-dependent manner. Accordingly, the recorded potentials of the three types of NPs in this study will open the door for their in vivo clinical evaluation.

## Data Availability

All data generated or analyzed during this study are included in this published article.
